# A Procedure for Taking a Remotely Controlled Elevator with an Autonomous Mobile Robot Based on 2D LIDAR

**DOI:** 10.3390/s23136089

**Published:** 2023-07-01

**Authors:** Jordi Palacín, Ricard Bitriá, Elena Rubies, Eduard Clotet

**Affiliations:** Robotics Laboratory, Universitat de Lleida, Jaume II, 69, 25001 Lleida, Spain

**Keywords:** elevator, lift, mobile robot, ICP, SLAM, multistory navigation, remote control

## Abstract

Navigating between the different floors of a multistory building is a task that requires walking up or down stairs or taking an elevator or lift. This work proposes a procedure to take a remotely controlled elevator with an autonomous mobile robot based on 2D LIDAR. The application of the procedure requires ICP matching for mobile robot self-localization, a building with remotely controlled elevators, and a 2D map of the floors of the building detailing the position of the elevators. The results show that the application of the procedure enables an autonomous mobile robot to take a remotely controlled elevator and to navigate between floors based on 2D LIDAR information.

## 1. Introduction

The autonomous navigation of a mobile robot across the different floors of a multistory building has many potential applications as extending last mile delivery [1,2], improved service robot assistance to humans [3,4], and surveillance [5,6]. Multistory building navigation presents several challenges related to the use of or interaction with elevators. These include identifying the current floor, locating and arriving at the elevator, calling it, entering through the narrow doorway, selecting the destination floor, and verifying arrival at the destination floor. The interaction with an elevator can be addressed using its original buttons and displays or, if available, using remote access or control.

Home or office service mobile robots [3,7,8,9,10,11] designed to perform tasks in multistory buildings have the drawback that most elevators are not equipped with remote controllers compatible with mobile robot usage. In some cases, upgrading the elevators may be impractical or too expensive. Then, the use of the original buttons of elevators represents a challenge for mobile robots as this involves many uncontrolled environmental factors that affect external recognition of the control panel, internal localization within the elevator car, and correct actuation of the buttons. These challenges are exacerbated by the large variety in control panel and button designs, the lack of easy-to-recognize features, and the fact that most elevators are built using reflective materials, such as polished steel and mirrors, which impede adequate detection.

So far, most of the scientific literature has been focused specifically on the challenging task of operating an elevator through the use of the original button panel, just as a human would do. For example, Yu et al. [12] used template-matching features for elevator-button detection. Klingbeil et al. [13] focused on the button recognition problem by developing a system that can detect, locate, and label the various controls in an elevator using vision algorithms combined with machine learning techniques. They trained the system with 150 images of 60 different elevators and performed offline tests with a success rate of 86.2%. The algorithm was then tested in a real environment with the STanford AI Robot (STAIR) mobile platform. The buttons were pressed using its five-DOF robotic arm with the aid of a camera equipped with depth sensors. All the buttons were identified correctly in the 14 tests performed, which took place in three different elevators that had not been used during the training phase of the algorithm. After identification, the arm actuator was capable of pressing the correct button in 13 of the 14 attempts. In a similar approach, Wang et al. [14] used image processing and pattern recognition techniques to detect the elevator buttons and to calculate the angle of each articulation of a custom-built robotic arm.

Kang et al. [15] proposed the use of image processing and pattern recognition strategies for button recognition and elevator status identification. This algorithm included a neural network to reject ambiguous candidates and to identify the call button, the destination floor buttons, the elevator’s current floor, and its moving direction. For navigating in an out of an elevator, they first generated an occupancy grid map of its interior to determine the safest location for the robot. This approach was tested in multiple simulated and real scenarios with successful results.

Troniak et al. [16] used a PR2 semi-humanoid omnidirectional robot created by Willow Garage that embedded two arm-like manipulators, cameras in the head and arm joints, and a variety of sensors including LIDAR and inertial measurement units. In this case, button detection was achieved using the head cameras and a template-matching algorithm based on previous knowledge of the object to be detected. The location of the buttons in the 3D space was performed using a textured-light projector combined with a stereo vision system. Once the buttons were identified and located, the mobile robot calculated a motion routine for its arms in order to push the buttons.

Abdulla et al. [17] proposed a procedure to detect the elevator control panel and its buttons by using a Kinect sensor in a semi-outdoor application. In this case, the effect of the incidence of direct sunlight was compensated by iteratively updating camera parameters and using manually attached fiducial points to define the area of interest for the visual button search. Then, optical character recognition (OCR) was used to identify the floor number and a custom robotic arm was guided to press the buttons.

Islam et al. [18] proposed elevator-button and floor-number recognition using a hybrid image classification approach based on the combination of a histogram of oriented gradients, bag-of-words models, and a feature selection algorithm based on an artificial neural network. Jiang et al. [19] proposed an improved two-stage deep neural network to locate and track the position of the panel buttons in real time from a mobile robot navigating autonomously using a 2D grid mapping approach. This approach was successfully tested in a multistory building using a prototype robot that detected and recognized the elevator buttons in challenging environments.

More recently, Manzoor et al. [20] compared the application of the network models You Only Look Once (YOLO) v3-tiny and v4-tiny for the task of elevator button recognition using different machine learning metrics applied to experimental results. In this case, the results showed promising overall accuracies of 97.91% and 98.60% using a 0.5 intersection over union (IoU) metric. In contrast with these proposals, this paper proposes an alternative approach to this problem assuming that an elevator is a fixed infrastructure that can be automated to simplify mobile robot interaction. The motivation is to address the problem of multistory building navigation with autonomous mobile robots.

### New Contribution

The new contribution of this work is the definition of a procedure to take a remotely controlled elevator with an autonomous mobile robot using 2D LIDAR. This work is inspired by the work of Jiang et al. [19], who proposed a procedure for entering an elevator with a mobile robot based on LIDAR and 2D grid mapping. The new procedure proposed in this work requires a mobile robot with a 2D LIDAR, a procedure for mobile robot self-localization, a building equipped with remotely controlled elevators using sliding doors, and a 2D map of the floors of the building detailing the position of each elevator. The experimental application of this procedure shows that an autonomous mobile robot can take a remotely controlled elevator and navigate between floors based on 2D LIDAR information. A video showing the application of this procedure is provided in [21].

The paper is structured as follows. Section 2 describes the materials and methods. In Section 3, the new procedures proposed are presented in detail. Section 4 presents the experimental results obtained in a real application with an autonomous mobile robot. Final remarks are given in Section 5.

## 2. Materials and Methods

The materials and methods used in this work are the APR-02 mobile robot, the method used for mobile robot self-localization, the 2D map of the floors of the building detailing the position of the elevators, and a remotely controlled elevator.

### 2.1. APR-02 Mobile Robot

The mobile platform used in this work is the APR-02 mobile robot, a three-wheeled, human-size, omnidirectional mobile robot developed at the Robotics Laboratory of the University of Lleida (Spain) [10]. This mobile robot is able to create the map of a building based on 2D LIDAR [22,23], operate autonomously in unconstrained indoor scenarios [24,25,26], and track any trajectory defined on a 2D map [22].

**Figure 1:**
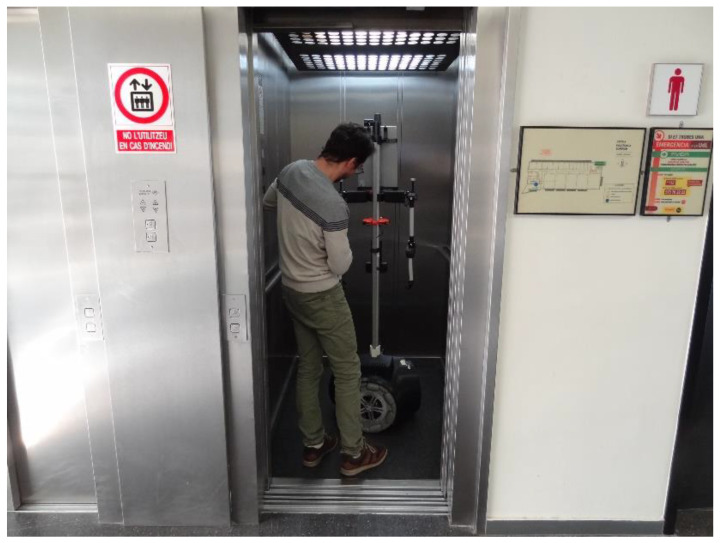
Image of one of the authors of the work assisting the APR-02 mobile robot in entering an elevator.

The APR-02 mobile robot has been deployed as an ambient monitoring tool to supervise temperature, humidity, and luminance [27] and as a walk-helper tool [28]. The main limitation of this robot was its incapacity to navigate between the different floors of a building without the help of an operator. Figure 1 shows one of the authors of the work assisting the APR-02 mobile robot in entering an elevator. The main problems detected when assisting the robot into an elevator were the need to prevent the door from closing automatically, robot guidance to enter and exit the elevator, and the need to enter the elevator at a certain velocity to prevent any wheel from getting stuck in the gap at the entrance or the door rails. The motivation of this work was to automate this assisted procedure in order to allow the mobile robot to autonomously navigate between floors in a multistory building.

### 2.2. Mobile Robot Self-Localization Based on 2D LIDAR

The development of multistory navigation requires a method for mobile robot self-localization based on processing the 2D LIDAR information. This 2D self-localization can be based on grid methods [19] or in point cloud methods [29,30]. The APR-02 mobile robot uses a high-performance Hokuyo UTM-30LX 2D LIDAR, based on an internal rotating laser range sensor that provides a planar 2D exploration (or scan) of the area around the sensor. This 2D LIDAR has a radial distance range of up to 40 m with a precision between 30 and 50 mm, covers 270° around the sensor, and provides 1081 points per scan at up to 40 scans/s.

**Figure 2:**
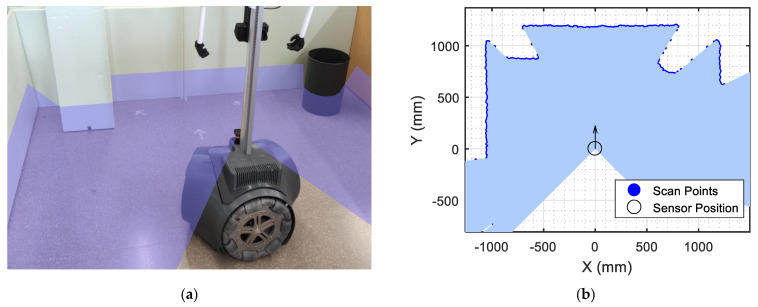
Information gathered by the 2D LIDAR of the APR-02 mobile robot: (**a**) representation of the scan plane; (**b**) real point cloud provided by the 2D LIDAR. The obstacle-free area is depicted with a transparent blue.

Figure 2a shows the mobile robot in a room in which the measurement plane of the 2D LIDAR scan has been manually colored with a transparent blue for easier interpretation. Complementarily, Figure 2b shows the scan gathered by the 2D LIDAR in which the blue area again depicts the obstacle-free area around the sensor and the blue points depict the walls and objects detected around the robot. This 2D LIDAR scan T can be described as follows:

(1) $$T=\left\{d_{1},\ldots ,d_{L}\right\}$$

where $d_{i}$ are the 2D distance points measured and L is the number of distance points in the scans (1081 points in the case of the UTM-30LX used in this work).

The APR-02 mobile robot uses the Iterative Closet Point (ICP) algorithm [29] to iteratively match the point clouds defined by the current scan provided by the 2D LIDAR $T_{p}$ and a 2D reference scan M. This reference scan M can be a detailed 2D map of the floor or, when a map is not available, a scan obtained previously (${M=T}_{p-1}$). This reference scan can be expressed as follows:

(2) $$M=\left\{r_{1},\ldots ,r_{K}\right\}$$

where $r_{i}$ are the 2D distance points included in the reference scan and K is the number of scan points. Compared with grid-based methods, the use of a point cloud approach for self-localization offers superior precision when using a high-precision 2D or 3D LIDAR [30] because it avoids the rounding caused by the use of a grid. In general, the number of points in T is always small and constant, while that in M is usually very large.

The ICP matching algorithm [29] iteratively searches for the best matching of each point $d_{j}$ (of T) in M by calculating the transformation $\left(R,t\right)$ that minimizes the Euclidean distance between these points:

(3) $$E{\left(R,t\right)}_{p}=\sum_{i=1}^{K}\sum_{j=1}^{L}\omega_{i,j}{\left\|r_{i}-\left(Rd_{j}+t\right)\right\|}^{2}$$

where $\omega_{i,j}$ are the weights of a point-to-point match, assigned as $\omega_{i,j}=1$ if $r_{i}$ is the closest point to $d_{j}$ and $\omega_{i,j}=0$ if $d_{j}$ has no matching point in M.

**Figure 3:**
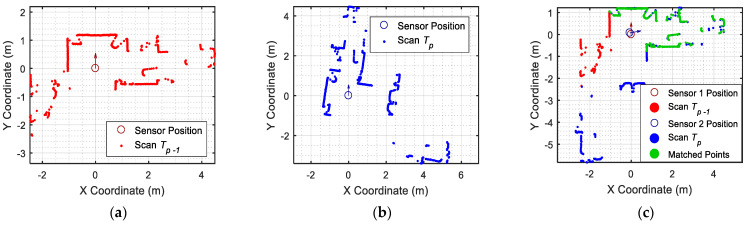
Representation of the results of the ICP matching algorithm: (**a**) reference scan with $M=T_{p-1}$; (**b**) current scan $T_{p}$; and (**c**) application of the transformation $\left(R,t\right)$ to $T_{p}$ to combine both scans and to create a new reference scan M; the matched points are depicted in green.

The transformation $\left(R,t\right)$ describes the relative displacement of the robot in M and the transformation required to project T in M. Figure 3 illustrates the application of the ICP matching algorithm [29]: Figure 3b shows the P’th scan $T_{p}$ provided by the 2D LIDAR of the mobile robot; Figure 3a shows the reference scan M which, in this simplified example, is a scan gathered previously (${M=T}_{p-1}$); and Figure 3c shows the projection of T relative to the reference scan M, which also describes the displacement and rotation of the robot between scans. The green points in Figure 3c highlight the matched or shared points between the point clouds T and M. 

The implementation of the ICP matching algorithm [29] in the APR-02 mobile robot is based on the Library for Iterative Closest Point Matching (LIBICP) implemented by Geiger et al. [30]. This was developed to register the ground truth point clouds used for evaluating the performance of stereo and optical flow systems. This implementation takes advantage of the optimized k-d tree search [31] provided in the C++ Boost library [32] to reduce the computational time required to find the nearest neighbors between two sets of point clouds based on point-to-point or point-to-plane matchings. The use of this library also allows for the application of the ICP matching when using 2D and 3D LIDAR [33], and hybrid sensor systems [34].

### 2.3. Map of the Different Floors of the Building

The procedure proposed in this work to take an elevator are based on the availability of a 2D map of each floor of the building in which the position of each automatic elevator is indicated using two waypoints: one located outside and in front of the elevator door and one located inside the car. This reference 2D map was created using the ICP algorithm [29] in a specific exploration of the empty building in order to avoid the registration of dynamic obstacles, such as people, in the map [22].

**Figure 4:**
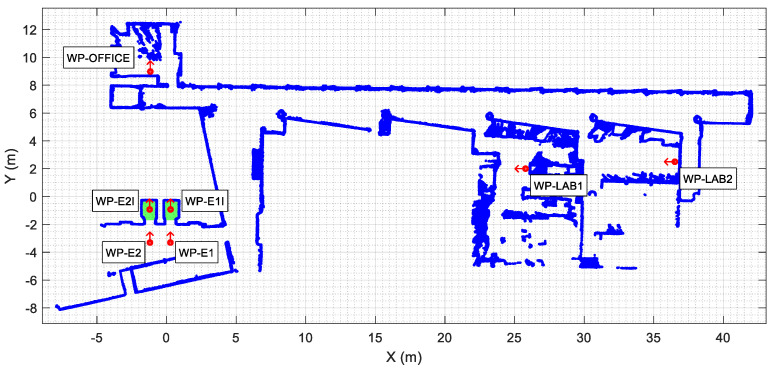
Point cloud map (blue points) obtained from the 2D scans registered on the second floor of the building. The waypoints labeled depict the localization (position as red circle and orientation as a red arrow) of some common destinations such as the elevators (green areas).

Figure 4 shows the point cloud describing the map used in this work. The map was created offline [35,36,37], combining the scans registered on the second floor of the Polytechnic School of the University of Lleida (Spain). The map depicts the reference point cloud M used in this work for mobile robot self-localization based on the ICP matching algorithm [29]. The map is also used to define the position of common destination waypoints and for path-tracking [22].

In this work, the map was manually edited [38] to define the following waypoints (WP) associated with the elevators: WP-E1 located in front of elevator 1, WP-E1I inside the car of elevator 1, WP-E2 in front of elevator 2, and WP-E2I inside the car of elevator 2. Additionally, the use of a mobile robot in a building requires the definition of common destination waypoints such as the main office, including a charging station (WP-OFFICE), and two laboratories (WP-LAB1 and WP-LAB2).

The point cloud map in Figure 4 has open sections in some areas enclosed with glass walls due to the measurement limitations of the infrared (IR) light used in 2D LIDARs. Floors 1, 2, and 3 of the building are identical, while floors 0 and −1 have some differences. However, the area that gives access to the elevators is identical on all floors. In this work focused on taking the elevator, the map in Figure 4 was used as a reference map M for all floors for the purpose of mobile robot self-localization.

### 2.4. Remotely Controlled Elevators

An elevator or lift is a machine that provides assisted vertical mobility. The use of the elevator in a multistory building requires the coordinated execution of a series of complex tasks: visual identification of the placement of the elevator; visual identification of the location of the buttons used to call the elevator; visual coordination of the motion of the arm and hand to press a specific button; visual identification of the opening of the elevator door; entering the elevator; visual identification of the buttons available to select the destination floor; visual coordination of the motion of the arm and hand in order to press a floor destination button; identification of the opening of the elevator door; verification of the destination floor before leaving the elevator; and exit from the elevator. Currently, the human-like implementation of the entire sequence of tasks required to interact with an elevator represents a challenge for autonomous mobile robots, and in general, they require assistance in order to use an elevator [39], with multistory building navigation proposed as future work [40].

The alternative to a human-like interaction with an elevator is to use a remote control either provided by the manufacturer or by the company responsible for maintenance. Unfortunately, in some cases, the remote control is not originally implemented or may not be suitable, affordable, or compatible with the software running on the mobile robots. The elevator used in this work did not have a remote control implemented and all the above-mentioned issues were assessed through the use of IoT device for elevator control proposed by Rubies et al. [41].

In general, most IoT devices used to monitor [42,43,44] and remotely control elevators [45,46] require the intrusive manipulation of both their wiring and mechanical structure. The advantage of the add-on IoT device used in this work [41] is that it is specifically designed to be attached over the original internal elevator button panel using servomotors to press the original buttons, without requiring any mechanical or electrical manipulation. When installed, the IoT device [41] provides remote access to the elevator through the pre-existing local area network (LAN) of the building. Table 1 shows the functions implemented in the mobile robot to control the elevators remotely. These functions wrappers the commands and sends messages to the IoT device that controls the elevator in order to press the original buttons on the elevator control panel [41].

**Figure 5:**
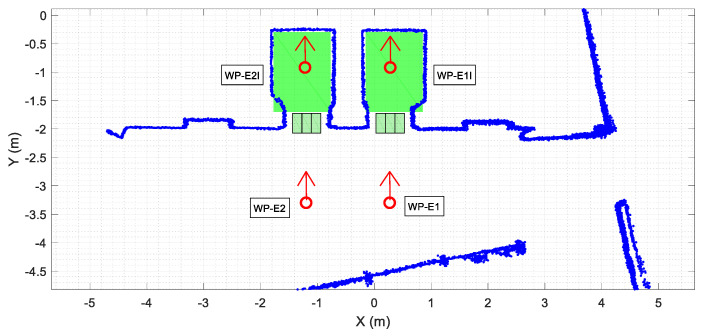
Detail of the area on the point cloud map (blue points) highlighting the waypoints used to define the position of the elevators (red circles). The inner area of the elevators is green, and the areas defined to detect the status of the doors are labeled with green rectangles.

As described above, the procedure proposed in this work to take the elevator requires accurate definition of the position of each elevator on the map (Figure 4) for each floor of the building. Figure 5 details the localization of the elevators and of these waypoints on the map of the building. The localization of elevator 1 is defined with two waypoints WP-E1 and WP-E1I. The first waypoint of elevator 1 (Figure 5, WP-E1) is located outside it, aligned with the center of its sliding doors, and oriented towards the door. Reaching the position and orientation of this waypoint will enable the mobile robot to enter the elevator car. The second waypoint for the elevator 2 (Figure 5, WP-E1I) is inside the car, aligned with the external waypoint in order to define a straight path (without maneuvers) from the outside to inside the elevator car. Similarly, the localization of elevator 2 is also defined with two waypoints WP-E2 and WP-E2I. The assumption is that a mobile robot reaching the external waypoints WP-E1 or WP-E2 will be ready to take a remotely controlled elevator and to navigate between floors although, in this work, only elevator 1 was optimized for use as a remotely controlled elevator.

Finally, the add-on IoT device used to control the elevators remotely does not provide information of the door status, so its state must be determined by the mobile robot. In this work, the doors are considered obstacles [47], and their state is detected via definition of three rectangular areas located across the area of the sliding doors. Figure 5 details the localization of these rectangular areas, which are implicitly defined by the localization of the elevator waypoints. When the mobile robot is in front of the elevator, the door state is established through the number of scan points detected inside these rectangular areas. 

## 3. Procedure to Take the Elevator

This section presents the procedure required to take a remotely controlled elevator based on 2D LIDAR. The task of taking the elevator is a single task containing a large sequence of steps or actions divided in two stages to improve their description: entering the elevator and exiting from it. 

### 3.1. Entering the Elevator

Table 2 shows a graphic interpretation, along with a short description, of the sequence of actions that the mobile robot must perform in order to board elevator 1 (or right) of the building safely. The small figures show the map (blue points) and the scan points (magenta points) gathered by the 2D LIDAR of the mobile robot, whose position and orientation are depicted with a green circle and thin line. The entering sequence starts by (1) setting the coordinates of the waypoint located in front of elevator 1 (WP-E1) as the target of the motion of the mobile robot. The next steps in the boarding sequence start when the mobile robot has arrived at this waypoint, requiring (2) sending the order to call the elevator to the IoT device controlling it; (3) waiting until the robot detects that the door of the elevator has begun to open; (4) sending the order to keep the door open to the IoT device of the elevator; (5) waiting until the robot detects that the door of the elevator is fully open; and (6) setting the coordinates of the waypoint inside the elevator (WP-E1I) as the new target location for the mobile robot trajectory and following the path to this waypoint. During this procedure, self-localization and path-tracking are performed by matching the current scan provided by the 2D LIDAR and the map of the floor. The entry procedure ends as soon as the mobile robot reaches the waypoint defined inside the elevator.

### 3.2. Exiting from Inside the Elevator

Table 3 shows a graphic interpretation, along with a short description, of the next sequence of actions that the mobile robot must perform in order to safely navigate between floors and exit elevator 1 (or right) of the building. This sequence starts assuming that the mobile robot has reached the waypoint defined inside the elevator (WP-E1I). Then, the next steps in the procedures are (7) sending the destination floor to the IoT device controlling the elevator; (8) sending the order to allow the elevator door to close to the IoT device; (9) rotating the mobile robot 180° in order to face the door of the elevator and waiting until it completes this rotation; (10) waiting until the door of the elevator being fully closed is detected; (11) waiting until the mobile robot detects that the door of the elevator is starting to open, assuming then that the mobile robot has arrived at its destination floor; (12) sending the order to keep the elevator door open to the IoT device; (13) setting the coordinates of the waypoint located outside the elevator (WP-E1) as the next target location for the mobile robot trajectory and following the path to this waypoint; and (14) reaching the waypoint defined outside the elevator and sending the order to allow the door to close to the IoT device controlling the elevator. At the end of this exiting procedure, the mobile robot is ready to continue navigating towards its destination.

### 3.3. Taking the Elevator

Table 4 shows the high-level function implemented in the APR-02 mobile robot to take a remotely controlled elevator. This function executes the procedures described sequentially in Table 2 and Table 3 to enter and exit the elevator using the external waypoint of the elevator (WP-E1) as a reference.

### 3.4. Path Planning including Navigation between Floors

The final step is to include the capability to navigate between the floors of the building in the procedure used to plan the path of the robot. As a comparative application example, Table 5 shows the high-level definition of two comparable mission plans: the single-floor mission is performed on one floor of the building and the dual-floor mission requires navigation between two different floors. The mission path can be defined using three high-level functions. The first defines the current or starting position of the mobile robot on the map, which is usually a charge station (SP1 and DP1). The second defines a destination (SP2, SP3, DP3, and DP5). The third is the procedure for taking the elevator and navigating between floors (DP2 and DP4). 

**Figure 6:**
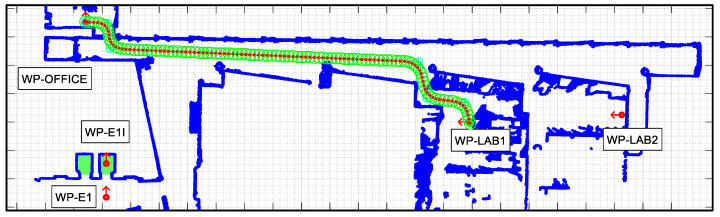
Path planned in the single-floor mission defined in Table 5 to go from WP-OFFICE to WP-LAB1 and from WP-LAB to WP-OFFICE.

**Figure 7:**
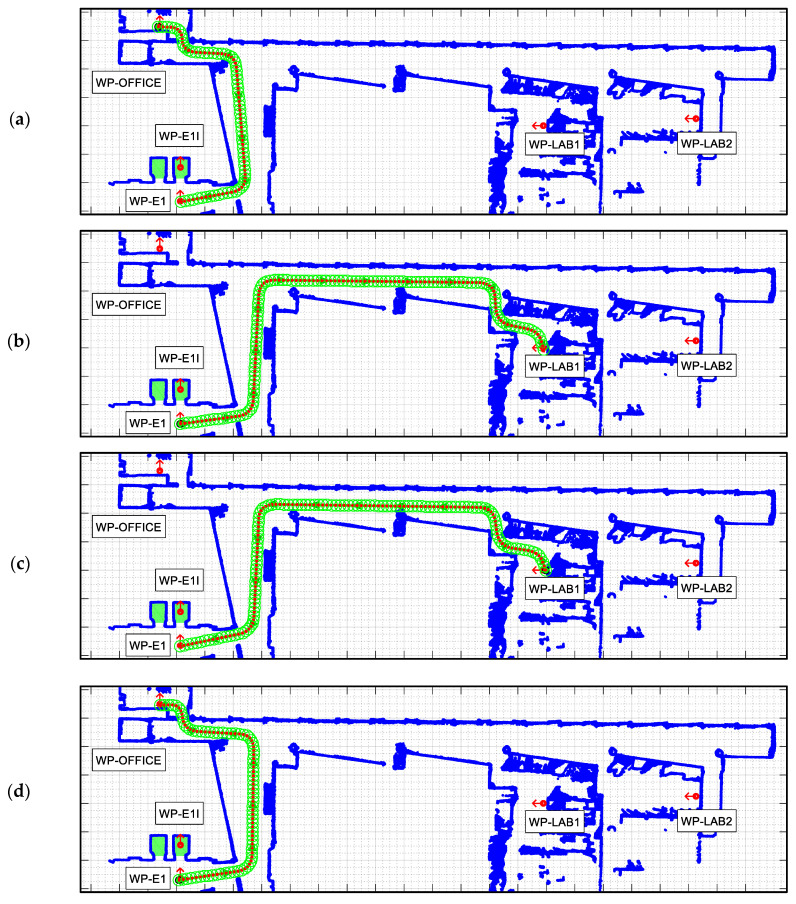
Paths planned in the dual-floor mission defined in Table 5 to go (**a**) from WP-OFFICE to WP-E1: (**b**) Floor 1: from WP-E1 to WP-LAB1 and (**c**) from WP-LAB1 to WP-E1, and (**d**) Floor 2: from WP-E1 to WP-OFFICE.

The APR-02 mobile robot is able to find the shortest path from its current position to a target position based on the implementation of the A* (A-star) search algorithm [48,49] and then follows this path until its intermediate or final destination is reached. The path-tracking procedure implemented in the APR-02 mobile robot to follow a path is based on the use of splines and the definition of a constant distance interval [50,51,52,53]. This procedure is described in [22], and its advantage is that it is not limited to the use of grids to define the trajectory of an omnidirectional mobile robot [54]. In the case of planning complex missions including the definition of several tasks, the definition of the optimal sequence of intermediate destinations may require prior optimization of the scheduling of the tasks, including the constraint of robot charging [55], for example, using genetic algorithms [56]. Figure 6 and Figure 7 summarize the path planned to carry out the single and dual-floor missions described in Table 5. Figure 6 shows the single-floor path computed by the APR-02 mobile robot to go from the waypoint WP-OFFICE to WP-LAB1 and to return, which, in this example, is the same path. In Figure 6 and Figure 7, the intermediate planned trajectory positions of the mobile robot are represented with green circles and the orientation of the robot in each position is represented with a red dot and a small red line. Comparatively, Figure 7 shows the dual-floor path computed to implement the same mission but with the waypoints located on two different floors of the building. In this case, the trajectory is represented with four illustrations: Figure 7a shows the trajectory from WP-OFFICE to WP-E1 on the second floor (this waypoint triggers the execution of the sequence of actions proposed to take the elevator); Figure 7b shows the trajectory on the first floor, from WP-E1 to WP-LAB1; Figure 7b shows the return to WP-E1 (in order to take the elevator again); and Figure 7c shows the return sequence to the second floor from WP-E1 to the starting point at WP-OFFICE.

## 4. Results

This section presents the results of the experiments conducted to validate the procedures proposed to take a remotely controlled elevator with an autonomous omnidirectional mobile robot.

### 4.1. Self-Localization Next to the Elevators

This subsection evaluates the mobile robot self-localization performances in the area of the elevators experimentally. The objective is to evaluate if the opening and closing of the automatic doors of the elevators affects the self-localization results obtained with the ICP [29] matching the map and the current scan provided by the 2D LIDAR of the mobile robot. For this evaluation, the APR-02 robot was positioned at the waypoint defined in front of elevator 1 (WP-E1).

**Figure 8:**
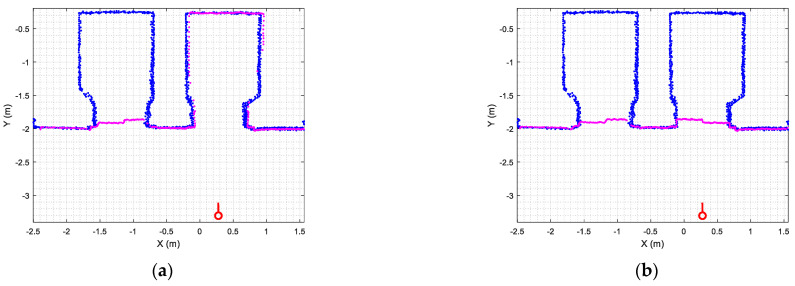
Matching between the map (blue points) and the 2D LIDAR scan (magenta points) provided by the mobile robot (red circle): (**a**) in the case of one elevator door closed; (**b**) in the case of both elevator doors closed.

Figure 8 illustrates two sample results of the ICP matching [29]. Figure 8a illustrates the matching between the map (blue points) with the doors open and the current 2D LIDAR scan provided by the mobile robot (magenta points) in the case of one door closed and Figure 8a in the case of both doors closed. Although there are discrepancies between the current scan (magenta points) and the map (blue points), these are not enough to cause the ICP algorithm to misidentify the position of the robot on the map. This is because the discrepancies originating by the closed doors (Figure 8a,b) are considered outliers and discarded during the ICP matching so they do not really affect the matching of both point clouds.

**Figure 9:**
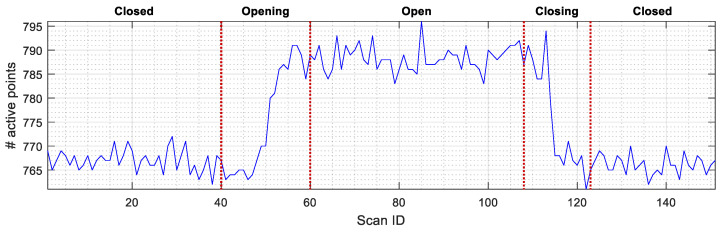
Evolution of the number of active points in the scans taken by the robot (obtained as a result of ICP matching) depending on the status of the door of elevator 1.

**Figure 10:**
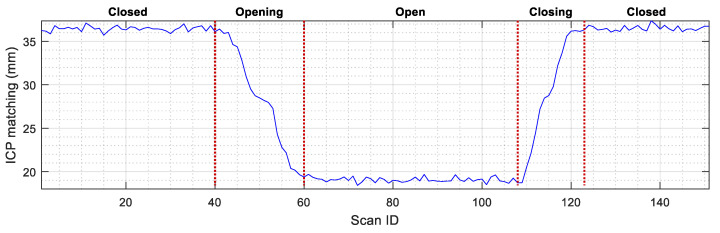
Evolution of the average inlier distance between the active points and the map (obtained as a result of ICP matching) depending on the status of the door of elevator 1.

Figure 9 and Figure 10 detail the discrepancies between the map of the floor (obtained with the doors open) and the scans taken by the robot, containing 1081 sample points in a 270° field of view from −135° to 135°. Figure 9 shows the evolution of the number of active points (scan points whose distance to the nearest map point is lower than a specific threshold) detected depending on the current state of the elevator door: closed, opening, closing, and closed. Figure 9 shows around 760 active (or matched) scan points when the door is closed, so there are around 321 unmatched scan points that have been identified as outliers, (see Figure 8a,b). Figure 9 shows that the number of active points gradually rises as the sliding door opens and the 2D LIDAR sensor detects the inner walls of the elevator. Once the door is fully open, the number of active points reaches 790. Alternatively, Figure 10 shows the evolution of the Euclidean distance that defines the cost function of the ICP matching algorithm [29], computed as the mean distance between the active points of the current scan (without outliers) and the points on the map. As expected, when the door is fully open, the differences between the scan taken by the robot and the map are minimal so the inlier average distance is significantly reduced. This can be further confirmed by performing a side-by-side comparison of Figure 9 and Figure 10, which shows an inverse relationship between number of active points and the mean inlier distance. 

**Figure 11:**
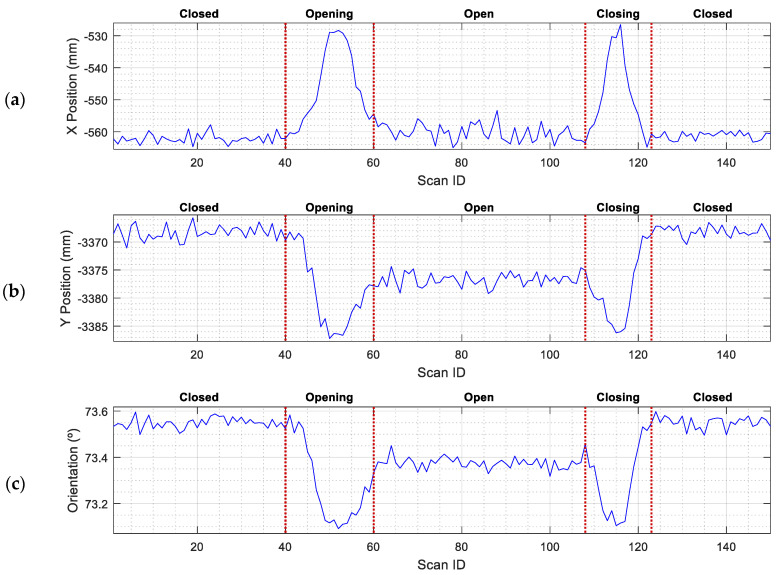
Evolution of the self-localization of the mobile robot (obtained as a result of ICP matching) depending on the status of the sliding door. The robot was in front of the elevator (at WP-E1): (**a**) X position variation; (**b**) Y position variation; (**c**) angular orientation variation.

Finally, Figure 11 shows the evolution of the self-localization of the robot depending on the status of the elevator door. Figure 11 indicates that the position of the mobile robot (x,y,θ) calculated with the ICP algorithm undergoes small variations when the door is in motion (opening or closing), mostly due to the variation in active points between the scans taken by the robot and the map. In general, the effect of opening and closing the door causes a position variation below 50 mm and an orientation error below 0.5°. Therefore, the conclusion of this experimentation is that the status of the doors does not interfere with the self-localization performances of the mobile robot.

### 4.2. Elevator Door Status Detection

This subsection experimentally evaluates the specific problem of detecting if the elevator doors are open, closed, or being opened or closed. The doors are considered as obstacles [47], and the detection of their state is performed by defining of three rectangular areas located across the area of the sliding doors. These three rectangular regions of interest are implicitly defined by the localization of the elevator waypoints, and the door status depends on the number of scan points counted inside. For this evaluation, the APR-02 mobile robot was positioned at the waypoint defined in front of elevator 1 (WP-E1).

**Figure 12:**
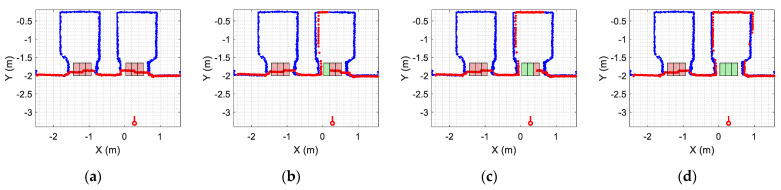
Depiction of the procedure used to detect the status of the sliding door of elevator 1. The mobile robot is stationary and in front of the elevator (at WP-E1) while the door is (**a**) fully closed; (**b**) one-third open; (**c**) two-thirds open; (**d**) fully open.

Figure 12 provides a set of snapshots depicting the different stages of the door status detection procedure while it is opening. The point cloud map is represented with blue dots, the point cloud of the 2D LIDAR scan gathered by the robot is represented with red dots, and the rectangular detection areas are labeled in green when there are no point clouds inside and in red when there are some points inside. Figure 12a shows that all the rectangular detection areas have scan points inside, so it is determined that the sliding door is closed. Figure 12b shows that one of the rectangular areas has no scan points, so the sliding door is starting to open. Figure 12c shows that two of the rectangular detection areas have no scan points inside, indicating that the sliding door is still opening. Figure 12d shows that no scan points are inside the three rectangular detection areas, indicating that the sliding door of the elevator is fully open. The experimental evaluation of this procedure proposed to detect the elevator door status based on the definition of three rectangular regions of interest was successful in all the experiments performed in front of or next to the elevators.

### 4.3. Path Tracking When Entering and Exiting the Elevator

This section presents the trajectory results obtained when entering and exiting elevator 1 of the Polytechnic School building in the University of Lleida (Spain) with the APR-02 mobile robot. In both cases, the reference point is the waypoint defined outside the elevator (WP-E1). Each mission experiment conducted was adapted to the performance of the motion system of the mobile robot [57,58], which in the case of the APR-02 mobile robot, is omnidirectional. This section is limited to the path tracking when entering and exiting the elevator because this trajectory is fundamental in taking the elevator while avoiding any collision with its door. 

Table 6 summarizes the experimental results obtained in 15 path-tracking validation experiments conducted when entering and exiting the elevator. Table 6 details the floor where the mobile robot was when it called the elevator, the destination floor, an evaluation of the navigation performed to enter and exit the elevator, and the floor where the robot exited the elevator. These results show that the robot had no self-localization or navigation problem when following the path required entering or exiting the elevator despite the small navigable area available.

**Figure 13:**
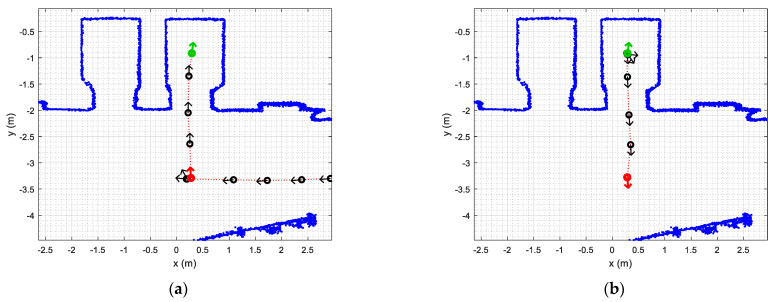
Ground truth mobile robot trajectory obtained from the 2D LIDAR scans in the cases of (**a**) entering the elevator, implementing the procedure described in Table 2; (**b**) exiting the elevator, implementing the procedure described in Table 3.

Figure 13a shows the results of one of the path-tracking experiments conducted. Figure 13a shows the ground truth trajectory followed by the APR-02 mobile robot arriving at the external waypoint of the first elevator (WP-E1, red circle) and then going to the inner elevator waypoint (WP-E1I, green circle). The task of entering the elevator was carried out by executing the sequence of actions described in Table 2. The straight trajectory defined in Figure 13a to go from the external elevator waypoint (WP-E1, red circle) to the internal elevator waypoint (WP-E1I, green circle) did not represent a challenge for the APR-02 mobile robot using a 2D LIDAR for self-localization and navigation. Similarly, Figure 13b shows the ground truth trajectory followed by the robot exiting the elevator. The task of entering the elevator was carried out by executing the sequence of actions described in Table 3. Again, the straight trajectory defined in Figure 13b to go from the internal elevator waypoint (WP-E1I, green circle) to the external elevator waypoint (WP-E1, red circle with the robot rotated 180 degrees) did not represent any challenge for the APR-02 mobile robot.

### 4.4. Taking the Elevator

Finally, this section experimentally evaluates the overall performance of taking a remotely controlled elevator with the autonomous mobile robot APR-02. Table 7 summarizes the experimental results provided in Table 6 in terms of successful and failed experiments. The objective of entering and exiting the elevator was executed successfully in all the validation experiments conducted. However, the objective of reaching a specific floor of the building failed in two experiments because the elevator stopped during its vertical trajectory to pick up other passengers who had called it. This cumulative or energy-saving picking behavior is normal in elevators located in multistory buildings and represented a problem because the IoT device controlling the elevator is not able to provide an estimation of the floor the elevator is on. Therefore, in some specific cases, the robot erroneously assumed that the floor destination had been reached. This is a limitation of the IoT add-on device used in this work to control the elevator remotely. This floor identification problem will be addressed in future improvements.

**Figure 14:**
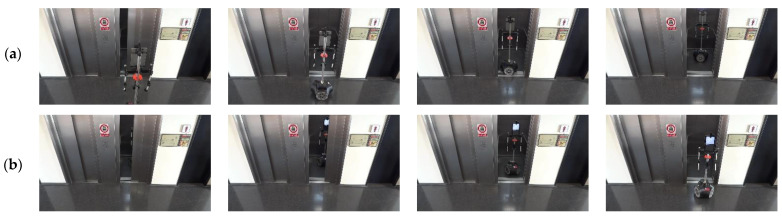
Sequence of images obtained when the mobile robot is taking an elevator: (**a**) entering the elevator; (**b**) exiting the elevator. A video showing these results is provided in [21].

In order to illustrate the results obtained, Figure 14 shows two sequences of images showing the APR-02 mobile robot entering and exiting the elevator on different floors of the building. These images are snapshots taken from the video provided in [21]. The most critical part of the process of entering and exiting in the elevator is to ensure that the elevator door does not close when the mobile robot is entering or exiting. In order to guarantee communication between the mobile robot and the IoT device controlling the elevator, the network messages submitted to the IoT device are based on the transmission control protocol (TCP) because it verifies the reception of messages, automatically resends them in case of occasional network communication errors, and warns in case of no handshake between the sender and the receiver because of persistent network communication errors [59,60]. The use of the TCP protocol during this communication guarantees control of the door of the elevator, allowing the mobile robot to enter and exit the elevator safely. 

## 5. Discussions and Conclusions

This work proposes a procedure for taking a remotely controlled elevator with an autonomous mobile robot based on the information provided by a 2D LIDAR. This approach extends the 2D grid mapping procedure proposed by Jiang et al. [19] to enter an elevator with a mobile robot using a 2D LIDAR. The implementation of the procedure requires a mobile robot with a 2D LIDAR, a remotely controlled elevator, a 2D self-localization method, and a 2D map of the floors of the building detailing the position of each elevator using two map waypoints: one located outside and in front of the elevator door, and one located inside its car.

The procedure was validated experimentally by conducting several experiments in a multistory building with five floors and two elevators. In this application case, the remote control of one elevator was provided by an IoT device [41] that enabled a mobile robot to call the elevator and to select a destination floor. This device has the advantage of not requiring any intrusive elevator manipulation as it is placed over the original button panel. 

A first experiment was designed to evaluate the effect of the elevator door status in the self-localization of the mobile robot. The results show that the opening status of the doors of the elevators does not affect the self-localization of the mobile robot around the area of the elevators. This is because the discrepancies between the 2D LIDAR scans and the 2D map caused by the door status are discarded as outliers during a self-localization procedure based on ICP matching [29].

A second experiment was designed to obtain the opening status of the elevator door. The results show that the opening status can be estimated successfully by applying obstacle detection techniques consisting of the definition of three virtual rectangular areas of interest inside the area of the door. Then, door status can be evaluated by monitoring the scan points inside these areas: a closed door causes points to appear inside these areas while there are no points when the door is open.

A third validation experiment was designed to evaluate the mobile robot following the trajectory required to enter and exit the elevator. The path used in the experiments is the straight trajectory defined between the internal and external elevator waypoints that are precisely located on the map of the floor. The results show that the path-tracking of this trajectory does not represent a challenge for a mobile robot implementing self-localization based on 2D LIDAR information. These good path-tracking results agree with the experimental results obtained by Kang et al. [15] and Jiang et al. [19] using an occupancy grid map to navigate in an elevator. In this case, the use of a point cloud map instead of a grid map has the advantage of providing an enhanced self-localization because grid discretization is avoided. 

Finally, the complete procedure proposed for taking a remotely controlled elevator in a building has been executed successfully in 15 validation experiments. Specifically, the goal of reaching a particular floor was achieved in the vast majority of the cases, but two experiments failed because the IoT device used in this work to control the elevator does not provide feedback about which floor the elevator is on. As a consequence of this limitation, the mobile robot can confuse an intermediate stop to pick up other passengers with a destination stop. However, this feedback limitation should not be expected in the case of a building with modern elevators.

The conclusion of this work is that an autonomous mobile robot can take a remotely controlled elevator and can navigate between floors based on the information gathered with a 2D LIDAR. A video showing the APR-02 mobile robot taking the elevator is provided in [21].

Future work will address the automatic estimation of the floor which the elevator is on and the shared and optimized use of elevators. Future work will also undertake the automatic detection and localization of elevators in unknown scenarios, the analysis of the navigation of an autonomous mobile robot in multistory buildings, and the implementation of multistory navigation for factory automatization [61].

## Figures and Tables

**Table 1:** Functions implemented in the mobile robot to control elevators remotely using the add-on IoT device proposed by Rubies et al. [41].

Function	Description
send_elevator (ID, FLOOR)	ID: is the identification of the elevator. FLOOR: is the destination floor of the elevator. In this work, the valid floors are: −1, 0, 1, 2 and 3.
send_elevator (ID, ACTION)	ID: is the identification of the elevator. ACTION: is an action implemented in the original button panel of the elevator. In this work, the valid actions are: KeepOpen, maintain the button that keeps the door open pressed. Close, releases the button that keeps the door open. Alarm, press the alarm button of the elevator.

**Table 2:** Procedure to enter elevator 1.

Sequence: Function	Description	Map (Blue) + LIDAR (Red)
	START of the procedure to enter elevator 1 (E1)	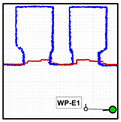
1: navigate_to (WP-E1)	The mobile robot must navigate to the waypoint located in front of elevator 1 (WP-E1).	
	The mobile robot reaches the waypoint located in front of elevator 1 (WP-E1) and is facing the door.	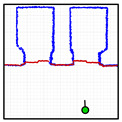
2: send_elevator (E1, Floor2)	The mobile robot calls the elevator (E1) from floor 2 (where the mobile robot currently is).	
3: waitfor_door_open (E1)	The mobile robot waits until it detects that the sliding door of the elevator is starting to open.	
	The sliding door of elevator 1 is detected as opening (green area without scans).	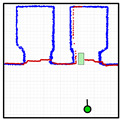
4: send_elevator (E1, KeepOpen)	The mobile robot sends elevator 1 the order to keep the door open in order to prevent unexpected door closing.	
5: waitfor_door_fullyopen (E1)	The mobile robot waits until the door of the elevator is fully open.	
	The mobile robot detects full opening of the sliding door (all three green areas in the door area without scan points).	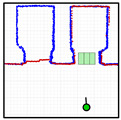
6: navigate_to (WP-E1I)	The mobile robot navigates to the waypoint inside the elevator (WP-E1I).	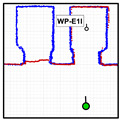
	The mobile robot reaches the waypoint located inside elevator 1 (WP-E1I).	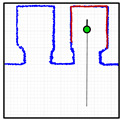
	The mobile robot is inside elevator 1 (E1) END of this partial sequence	

**Table 3:** Procedure to exit from inside elevator 1.

Sequence: Function	Description	Map (Blue) + LIDAR (Red)
	START of this partial sequence	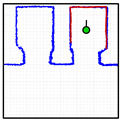
7: send_elevator (E1, Floor1)	Sends the elevator (E1) the destination floor	
8: send_elevator (E1, Close)	Allows automatic closing of the elevator door	
9: rotate (180°)	The mobile robot rotates 180° to exit	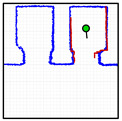
	The mobile robot completes the rotation	
10: waitfor_door_closed (E1)	The mobile robot waits until the door of the elevator is fully closed	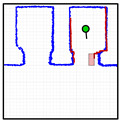
	The mobile robot detects full closure of the sliding door of the elevator	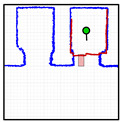
11: waitfor_door_open (E1)	The mobile robot waits for full opening of the door of the elevator	
	The mobile robot detects full opening of the sliding door	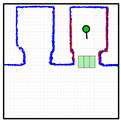
12: send_elevator (E1, KeepOpen)	Send elevator the order to keep the door open	
13: navigate_to (WP-E1)	The mobile robot navigates to the waypoint outside the elevator (WP-E1)	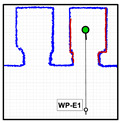
	The mobile robot reaches the waypoint outside the elevator (WP-E1)	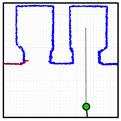
14: send_elevator (E1, Close)	Allow automatic closing of the door	
	END of the procedure to take elevator 1 CONTINUE navigation	

**Table 4:** Function implemented to take elevator 1.

Function	Description
goto_floor (FLOOR)	FLOOR: is the destination floor of the mobile robot. In this work, the valid floors are −1, 0, 1, 2 and 3. This macro function defines the external waypoint of elevator 1 (WP-E1) as the new mobile robot trajectory destination, enters and exits from the elevator, and ends with the mobile robot located at (WP-E1) on the specified destination FLOOR (sequences in Table 2 and Table 3)

**Table 5:** Definition of two comparable mission plans performed on the same floor and requiring navigating between floors.

Single-Floor Mission Sequence: Function	Dual-Floor Mission Sequence: Function
SP1: start_at (Floor2, WP-OFFICE) SP2: move_to (WP-LAB1) SP3: move_to (WP-OFFICE)	DP1: start_at (Floor2, WP-OFFICE) DP2: goto_floor (Floor1) DP3: move_to (WP-LAB1) DP4: goto_floor (Floor2) DP5: move_to (WP-OFFICE)

**Table 6:** Description of the experiments conducted in this work to navigate between floors.

Experiment	Starting Floor	Destination Floor	Navigation Problem	Arrival Floor
1	2	1	No	1
2	1	2	No	2
3	2	3	No	3
4	3	−1	No	0 ^1^
5	0	2	No	2
6	2	0	No	0
7	0	3	No	3
8	1	0	No	0
9	0	3	No	2 ^1^
10	2	−1	No	−1
11	−1	0	No	0
12	0	2	No	2
13	2	1	No	1
14	1	3	No	3
15	3	2	No	2

^1^ The elevator stopped at an intermediate floor to pick up another passenger.

**Table 7:** Experimental results obtained when the APR-02 mobile is taking an elevator.

Concept	Number of Experiments	Successful Experiments	Failed Experiments	Success Rate
Entering the elevator	15	15	0	100%
Exiting the elevator	15	15	0	100%
Arriving at the planned destination floor	15	13	2 ^1^	86%

^1^ The elevator stopped at an intermediate floor to pick up another passenger.

## Data Availability

Not applicable.
